# Knowledge of Antiretroviral Treatment and Associated Factors in HIV-Infected Patients

**DOI:** 10.3390/healthcare9040483

**Published:** 2021-04-20

**Authors:** Lam Van Nguyen, Thao N. P. Nguyen, Anh N. Thach, Anh N. Lam, Duc Q. Lam, Chu X. Duong, Suol T. Pham, Thao H. Nguyen, Dyah A. Perwitasari, Katja Taxis, Phuong M. Nguyen, Thang Nguyen

**Affiliations:** 1 Department of Surgery, Can Tho University of Medicine and Pharmacy, Can Tho City 900000, Vietnam; nvlam@ctump.edu.vn; 2 Department of Pharmacology and Clinical Pharmacy, Can Tho University of Medicine and Pharmacy, Can Tho City 900000, Vietnam; nnpt1997@gmail.com (T.N.P.N.); haoquang088@gmail.com (D.Q.L.); dxchu@ctump.edu.vn (C.X.D.); ptsuol@ctump.edu.vn (S.T.P.); 3 Soc Trang Center for Disease Control, Soc Trang Province 96000, Vietnam; anhpacst@gmail.com; 4 Department of Epidemiology, Can Tho University of Medicine and Pharmacy, Can Tho City 900000, Vietnam; lnanh@ctump.edu.vn; 5 Department of Clinical Pharmacy, University of Medicine and Pharmacy at Ho Chi Minh City, Ho Chi Minh City 710000, Vietnam; huongthao0508@gmail.com; 6 Faculty of Pharmacy, University of Ahmad Dahlan, Yogyakarta 55166, Indonesia; diahperwitasari2003@yahoo.com; 7 Groningen Research Institute of Pharmacy, University of Groningen, 9713 Groningen, The Netherlands; k.taxis@rug.nl; 8 Department of Pediatrics, Can Tho University of Medicine and Pharmacy, Can Tho City 900000, Vietnam; nmphuong@ctump.edu.vn

**Keywords:** knowledge, HIV/AIDS, ARV, Vietnam

## Abstract

This study aimed to assess the knowledge of antiretroviral (ARV) treatment and the associated factors in HIV-infected patients in Vietnam. We conducted a cross-sectional descriptive study of 350 human immunodeficiency virus (HIV)/acquired immunodeficiency syndrome (AIDS) patients being treated with ARV at outpatient clinics at Soc Trang, Vietnam, from June 2019 to December 2019. Using an interview questionnaire, patients who answered at least eight out of nine questions correctly, including some required questions, were considered to have a general knowledge of ARV treatment. Using multivariate logistic regression to identify factors associated with knowledge of ARV treatment, we found that 62% of HIV-infected patients had a general knowledge of ARV treatment, with a mean score of 8.2 (SD 1.4) out of 9 correct. A higher education level (*p* < 0.001); working away from home (*p* = 0.013); getting HIV transmitted by injecting drugs or from mother-to-child contact (*p* = 0.023); the presence of tension, anxiety, or stress (*p* = 0.005); self-reminding to take medication (*p* = 0.024); and a high self-evaluated adherence (*p* < 0.001) were found to be significantly associated with an adequate knowledge of ARV treatment. In conclusion, education programs for patients, as well as the quality of medical services and support, should be strengthened.

## 1. Introduction

From the discovery of the world’s first human immunodeficiency virus (HIV) infection in Los Angeles, USA, in 1981, until 2020, according to the Joint United Nations Programme on HIV/AIDS (UNAIDS), about 38 million people in the world have been affected with HIV; this includes 36.2 million adults and 1.8 million children [1]. Vietnam reported about 230,000 HIV cases, and 5000 acquired immunodeficiency syndrome (AIDS)-related deaths among patients of all ages [1]. In Soc Trang Province, as of 31 December 2020, the total number of HIV cases was 4333, including 1657 AIDS-related deaths [2].

Since 2005, the Vietnam Ministry of Health has expanded antiretroviral (ARV) services for HIV patients, as recommended by the World Health Organization [3]. Previous studies have indicated that patients with inadequate knowledge are 3.5 times more likely to default antiretroviral therapy (ART) and miss ART appointments compared with those with adequate knowledge [4]. Moreover, HIV patients’ lack of knowledge may be influenced by inadequate information provided by health professionals regarding medication [5]. This lack of knowledge can be a barrier to ART adherence [5,6], as it is closely related to patients’ health outcomes [7] and to the effectiveness of pharmacotherapy [8], including virological failure [8,9,10], antiretroviral resistance [8,9,10,11], and increased mortality [8,11]. Therefore, knowledge regarding ARV treatment must be provided effectively through counseling programs, as ART is one of the most common therapies available today. Until now, in Soc Trang Province, a resource-limited province in southern Vietnam, the treatment of HIV/AIDS patients at its AIDS Center has been a great challenge because of patients’ lack of knowledge about ARV treatment. However, having recently received considerable support in resources to carry out this work [12], we conducted this study in Soc Trang to assess HIV-infected patients’ knowledge of ARV treatment and associated factors.

## 2. Materials and Methods

At outpatient clinics of Soc Trang General Hospital from June 2019 to December 2019, we conducted a cross-sectional descriptive study on HIV-infected participants who were aware of their HIV status. We obtained ethical approval from Soc Trang General Hospital and the ethics boards of Can Tho University of Medicine and Pharmacy (approval no. 21/HDDD, dated 19 February 2019).

Our target population consisted of participants who were at least 18 years of age and had been receiving ART for 6 months or more. We excluded patients who were suffering from mental illness (determined by using the mini-mental state examination; respondents were unqualified if they had a score of 17 or less out of a maximum score of 30 [13]); also excluded were patients lost to follow-up or who had died during this study, as well as patients who refused to participate.

We determined the sample size of 334 patients using the formula $n=Z_{1-\alpha /2}^{2}\frac{p\left(1-p\right)}{d^{2}}$ (*n*: sample size; *Z*: Z-score value for standard normal distribution; *α*: type I error probability of 5%; *p*: the proportion in a previous study of 68%; *d*: tolerable sampling error of 5%). To avoid a specimen damage rate, we added approximately 5% more participants to the sample; thus, 350 participants were selected. The process of selection was as follows: from a total number of 861 HIV-infected patients being treated at the study site, we excluded all ineligible patients; we then applied a systematic random sampling method until the target of 350 participants was reached.

A previously used questionnaire about ARV treatment was modified to suit the culture and context of this study [14,15]. The questionnaire was approved by faculty members of Can Tho University of Medicine and Pharmacy, Vietnam. The questionnaire was divided into two parts. The first part collected demographic and ARV treatment characteristics. The second part was used to assess patients’ knowledge of ARV treatment, using nine questions related to drug classification, ARV combination regimens, treatment period, drug-taking, side-effects, management of side-effects, management of missed medication, calculation of following doses, and self-evaluated adherence. Questionnaire scores ranged from 0 to 9, with one point for each correct answer. Those who scored not less than eight out of nine, with correct answers to mandatory questions three to nine, were classified as having a general knowledge of ARV treatment. The questionnaire also included questions on medical services and support, to be explored in correlation with the above factors regarding ARV treatment knowledge.

Data were entered and analyzed using Microsoft Excel and SPSS version 20.0, respectively. The results were shown as frequency, percentage, odds ratio (OR), and 95% confidence interval (95% CI). We used univariable and multivariable analysis models to identify the factors associated with patients’ knowledge of ARV treatment.

## 3. Results

### 3.1. Demographic Characteristics

**Figure 1:**
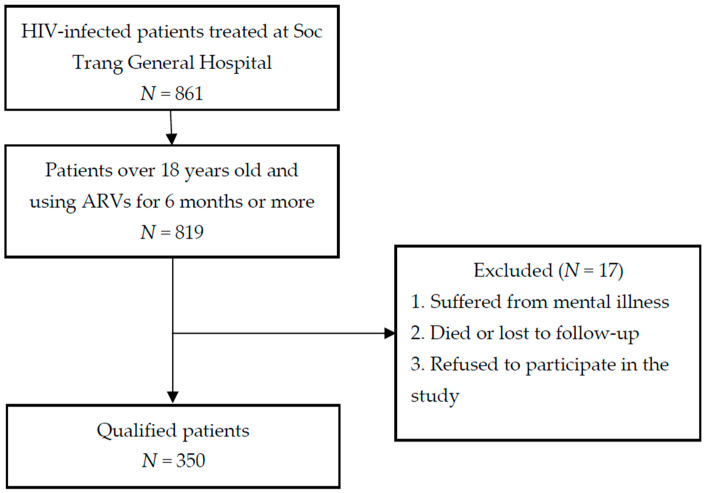
Enrollment of participants.

The flowchart of the study population is presented in Figure 1. Of 350 interviewed participants, 62.9% were male. The mean age was 35 years (SD 8.9), and the predominant age group was >35 years (53.7%). Of the participants, 58 % had at least a secondary school education, and most were employed (81.4%). The most commonly reported cause of HIV was unprotected sexual intercourse (95.1%). The majority of participants did not suffer from opportunistic infections (97.1%). Furthermore, 69.1% of participants reported that the distance to the clinic offering ARVs was greater than 20 km (Table 1).

### 3.2. Knowledge of ARV Treatment

As shown in Table 2, in which the correct answers are indicated in brackets, the proportion of patients with a general knowledge of ARV treatment was relatively high (62%), with a mean score of 8.2 ± 1.4 (SD) out of 9 correct. Most (>90%) of these participants were aware that ARVs are antiviral medicine, and were also aware of the side-effects of ARVs, management of side-effects, and the calculation of subsequent doses. Nevertheless, a small number reported that they did not know how to manage treatment after missing medication. Noticeably, 96.6% of participants gained knowledge of ARV adherence through the study.

### 3.3. Factors Associated with Knowledge of ARV Treatment

The univariable logistic regression revealed that better knowledge of ARV treatment was associated with a higher education level ((OR) = 3.25, 95% CI: 2.07–5.09; *p* < 0.001), working away from home ((OR) = 1.91, 95% CI: 1.21–3.03; *p* = 0.005), and getting HIV from injecting drugs or mother-to-child contact ((OR) = 4.86, 95% CI: 1.09–21.62; *p* = 0.022). Additionally, factors relating to medical services and support were significantly correlated with knowledge of ARV treatment (Table 1).

In the multivariable logistic regression, good knowledge of ARV treatment was significantly associated with an education level of secondary school or more ((aOR) = 2.54, 95% CI: 1.56–4.13), working away from home ((aOR) = 1.91, 95% CI: 1.14–3.18), and having HIV transmitted by injecting drugs or through mother-to-child contact ((aOR) = 6.65, 95% CI: 1.29–34.07). Patients experiencing tension, anxiety, or stress, and reminding themselves to take the medication tended to have better knowledge of ARV treatment ((aOR) = 2.04, 95% CI: 1.24-3.34 and (aOR) = 2.12, 95% CI: 1.1–4.06; respectively). In terms of self-evaluated adherence, patients with a high adherence had better knowledge than those with a medium or low adherence ((aOR) = 4.06, 95% CI: 2.17–7.57; Table 3).

## 4. Discussion

The majority of participants had adequate knowledge of ARV treatment (62%). The mean score was 8.2 of 9 correct, or 91%, which was higher than that in previous studies [6,16,17,18,19]. In our study, having a general knowledge of ARV treatment was defined as having a score of eight or more, and responding correctly to questions three to nine. This comparison is somewhat biased, as the score was assessed using a questionnaire that differed from those used in published research. However, our measurement tool also had strengths. First, it had previously been used and well-applied, as well as being highly appreciated, in other studies conducted in Vietnam [14,15]. Secondly, our modified questionnaire was a short and understandable version, which optimized the length of the interview and especially suited patients with poor literacy. However, it also had limitations—it was not validated or approved by any associations, and the reliability of its Cronbach’s alpha was not well evaluated.

Of the nine questions, the question regarding ARV combinations had the highest percentage of incorrect responses (28.3%). Most of the participants (97.1%) had been prescribed first-line therapy, which involved triple-ARV combination regimens based on WHO guidelines. However, health care workers were not focused on explaining this information, and patients were likely to forget this content of the counseling program after long-term therapy. Additionally, patients with poor literacy and educational levels may have had difficulties in understanding the complex and specific terminologies regarding essential factors of adherence [20,21].

Regarding what to do after missing medication, the number of patients who did not know what to do was relatively high (11.1%). However, our findings were still lower than those in other studies (20% and 85.6%, respectively) [21,22]. This information is necessary to help patients get better treatment outcomes and to avoid overdosing because of a double dose [21]. A lack of this knowledge can lead not only to suboptimal adherence rates, but also to high rates of drug-resistance [23], immunological failure [24], virological failure [25], and clinical failure [26]. These results indicated that interventions should concentrate on educating patients about how to deal with a missed dose.

ART is the only effective therapy for improving health conditions and prolonging life. Adequate adherence plays an important role in minimizing the prevalence of antiviral resistance and contributing to the success of the treatment. However, not all patients and caregivers are fully aware of this fact. Hence, they need to be equipped with adequate general knowledge of ARV throughout their treatment.

Multivariate analyses showed education level, working away from home, transmission routes, and factors of medical services and support to be statistically associated with patients’ level of ARV knowledge. In terms of educational level, our results support previous reports [4,16,23]. Patients having higher education levels had higher scores, as they were aware of their condition, and had a healthier lifestyle [16] and better understanding of medical knowledge [8] and the importance of treatment. On the other hand, being away from home was one of the reasons leading to missing doses [23] and non-adherence [27]. This finding made a valuable contribution our knowledge regarding adherence. It could be explained that patients had greater exposure to educational media and broadened their knowledge when they went to work in neighboring cities, compared with those who worked locally in a small province with poor socioeconomic status and low education level. Alarmingly, HIV patients infected by sexual intercourse were 6.65 times less likely than the others to have a general knowledge. Nevertheless, becoming aware of their status translated into decreased high-risk transmission behaviors [28]; those who had low knowledge scores also had poor attitudes towards sexual intercourse [29]. Previous research indicated that Vietnamese HIV patients with a high knowledge of how to prevent HIV transmission (>80%) [30], combined with their use of ART, did not increase high-risk transmission behaviors [28]; however, intensive HIV education programs should be provided for this group to increase their knowledge and positive attitude, and to reduce disease transmission and progression.

Our study has some limitations that need to be addressed. Our results may not be generalized to all HIV-infected patients in the nation, because the sample size was limited to patients from outpatient clinics in a rural province. Future research needs to investigate other factors and barriers affecting improvements in knowledge, as well as enhancing medical services and support. Better knowledge can be expected to maximize patient adherence to treatment.

## 5. Conclusions

Our data show that 62% of HIV-infected participants had a general knowledge of ARV treatment, with a mean score of 8.2 ± 1.4 (SD). Nevertheless, a proportion of patients reported that they did not know how to manage treatment after missing medication, and did not know ARV combinations. Patients with a higher education level, working away from home, getting HIV by injecting drugs or mother-to-child contact, were more likely to have better knowledge. Notably, satisfaction with medical services and support also had a positive impact on their knowledge. Interventions to further educate patients, as well as more adequate information and improved quality of medical services, are needed in order to provide necessary support and care when patients have problems related to their therapy, in order to maintain a good relationship with patients and to build strategies for counseling.

## Figures and Tables

**Table 1:** Study population characteristics: demography, antiretroviral (ARV) treatment, and medical services and support.

Characteristics	*n* (%)	General Knowledge of ARV Treatment (≥8 of 9 Answers)		Unadjusted	
		Yes *n* (%)	No *n* (%)	OR (95% CI)	*p*-Value *
**Age group**					
<35 years	162 (46.3)	106 (65.4)	56 (34.6)	1.31	0.219
≥35 years	188 (53.7)	111 (59.0)	77 (41.0)	(0.85–2.03)	
**Gender**					
Male	220 (62.9)	139 (63.2)	81 (36.8)	1.14	0.553
Female	130 (37.1)	78 (60.0)	52 (40.0)	(0.73–1.79)	
**Distance to clinic offering ARVs**					
<20 km	108 (30.9)	70 (64.8)	38 (35.2)	1.19	0.469
≥20 km	242 (69.1)	147 (60.7)	95 (39.3)	(0.74–1.91)	
**Education level**					
Secondary school or more	206 (58.9)	151 (73.3)	55 (26.7)	3.25	<0.001
Less than secondary school	144 (41.1)	66 (45.8)	78 (54.2)	(2.07–5.09)	
**Occupational status**					
Currently employed	285 (81.4)	183 (64.2)	102 (35.8)	1.64	0.074
Unemployed	65 (18.6)	34 (52.3)	31 (47.7)	(0.95–2.82)	
**Working away from home**					
Yes	135 (38.6)	96 (71.1)	39 (28.9)	1.91	0.005
No	215 (61.4)	121 (56.3)	94 (43.7)	(1.21–3.03)	
**HIV transmission routes**					
Injecting drugs or mother-to-child contact	17 (4.9)	15 (88.2)	2 (11.8)	4.86	0.022
Sexual intercourse	333 (95.1)	202 (60.7)	131 (39.3)	(1.09–21.62)	
**Opportunistic infections**					
No	340 (97.1)	212 (62.4)	128 (37.6)	1.66	0.514 *
Yes	10 (2.9)	5 (50.0)	5 (50.0)	(0.47–5.83)	
**Alcohol and tobacco use**					
No	106 (30.3)	71 (67.0)	35 (33.0)	1.36	0.206
Yes	244 (69.7)	146 (59.8)	98 (40.2)	(0.84–2.20)	
**Presence of tension, anxiety, or stress**					
Yes	160 (45.7)	115 (71.9)	45 (28.1)	2.21	<0.001
No	190 (54.3)	102 (53.7)	88 (46.3)	(1.41–3.45)	
**Patients’ quality of life after treatment**					
Better	239 (68.3)	164 (68.6)	75 (31.4)	2.39	<0.001
Normal	111 (31.7)	53 (47.7)	58 (52.3)	(1.51–3.80)	
**Patients wishing to stop medication after symptoms improved**					
No	294 (84)	193 (65.6)	101 (34.4)	2.55	0.001
Yes	56 (16)	24 (42.9)	32 (57.1)	(1.43–4.56)	
**Reminder support to take ARVs**					
Self-remind	293 (83.7)	193 (65.9)	100 (34.1)	2.65	0.001
Spouses, parents, siblings	57 (16.3)	24 (42.1)	33 (57.9)	(1.49–4.73)	
**Self-evaluated adherence**					
High adherence	282 (80.6)	194 (68.8)	88 (31.2)	4.31	<0.001
Medium and low adherence	68 (19.4)	23 (33.8)	45 (66.2)	(2.46–7.57)	

* Fisher’s Exact Test.

**Table 2:** Knowledge of ARV treatment.

Question	Frequency (*n* = 350)	Percentage (%)
**1. What are antiretroviral drugs?**		
Antivirals (correct)	320	91.4
Antibiotics/do not know	30	8.6
**2. How many types of drugs are in the ARV combinations?**		
Triple ARVs or more (correct)	251	71.7
Do not know	99	28.3
**3. How long does treatment last?**		
Lifelong (correct)	341	97.4
Other duration/do not know	9	2.6
**4. How does one take ARVs correctly?**		
Once daily (q.24.h) or twice daily (q.12.h) (correct)	343	98.0
Do not know	7	2.0
**5. What are the side-effects of taking ARVs?**		
Rash, headache/dizziness, nausea/vomiting, diarrhea, stomachache, anemia, hepato-renal toxicity, etc. (correct)	334	95.4
Do not know	16	4.6
**6. How to manage the side-effects of taking ARVs?**		
Consult with physicians (correct)	320	91.4
Self-manage/do not know	30	8.6
**7. How to manage after missing medication?**		
Take it as soon as you remember (correct)	311	88.9
Skip the missed dose/do not know	39	11.1
**8. How to calculate the next doses?**		
Take the next dose 4 h after taking the missed dose (for the twice-daily regimen) or 12 h (for the once-daily regimen) (correct)	328	93.7
Do not know	22	6.3
**9. What is treatment adherence?**		
Take the right medicine, right dose, at the right time, in the right way, and re-examine on time (correct)	338	96.6
Do not know	12	3.4
**General knowledge of ARV treatment (≥ 8 of 9 scores)**	217	62.0
**Mean score (SD): 8.2 (1.4)**		

**Table 3:** Factors associated with knowledge regarding ARV treatment.

Covariate	Adjusted	
	OR (95% CI)	*p*-Value
**Education level**		
Secondary school or more	2.54 (1.56–4.13)	<0.001
Less than secondary school		
**Working away from home**		
Yes	1.91 (1.14–3.18)	0.013
No		
**HIV transmission routes**		
Injecting drugs or mother-to-child contact	6.65 (1.29–34.07)	0.023
Sexual intercourse		
**Patients with tension, anxiety, or stress**		
Yes	2.04 (1.24–3.34)	0.005
No		
**Reminder support to take ARVs**		
Self-remind	2.12 (1.1–4.06)	0.024
Spouses, parents, siblings		
**Self-evaluated adherence**		
High adherence	4.06 (2.17–7.57)	<0.001
Medium and low adherence		

## Data Availability

Data are contained within the article. Data sharing is not applicable to this article.
